# On Controls in Ancient Microbiome Studies, and Microbial Resilience in Ancient Samples

**DOI:** 10.3390/genes9100471

**Published:** 2018-09-27

**Authors:** Tasha M. Santiago-Rodriguez, Gary A. Toranzos

**Affiliations:** 1 Diversigen Inc., Houston, TX 77046, USA; 2 Environmental Microbiology Laboratory, Biology Department, University of Puerto Rico, San Juan, PR 00932, USA; gary.toranzos@upr.edu

**Keywords:** blank controls, microbiome, mummies, preservation

## Abstract

In the following comment, we reply to Eisenhofer and Weyrich’s letter “Proper authentication of ancient DNA is still essential” responding to the article “Gut Microbiome and Putative Resistome of Inca and Italian Nobility Mummies” by Santiago-Rodriguez et al. One of the concerns raised was the possibility that the patterns noted in the gut microbiome of pre-Inca/Inca and Italian nobility mummies were due to contamination of the blank control. When examining the blank controls and filtering the operational taxonomic units (OTUs) present in the blank controls, and further performing in-silico contamination analyses, we noticed very similar patterns as those previously reported. We also discuss controls in ancient microbiome studies, and aspects of microbial resilience in ancient samples.

## 1. Controls in Ancient Microbiome Studies

**Figure 1:**
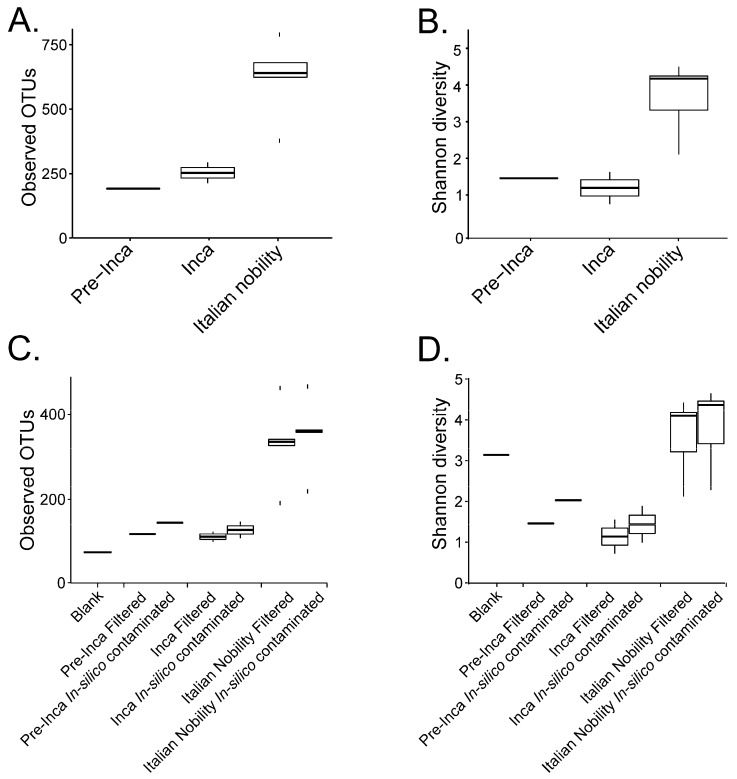
Alpha diversity of the mummified gut remains. Results show the alpha diversity values of the pre-Inca/Inca and Italian nobility mummies. Observed OTUs (**A**) and evenness (Shannon diversity index) (**B**) were calculated after filtering OTUs present in the blank control from the mummified microbiome (see text). Sequence files were also in-silico contaminated with the OTUs identified in the blank control to mimic cross contamination of the samples with extraction blanks. Observed OTUs (**C**) and evenness (Shannon diversity index) (**D**) were also determined for the in-silico contaminated samples.

Several points should be addressed regarding Eisenhofer and Weyrich’s letter responding to the article “Gut Microbiome and Putative Resistome of Inca and Italian Nobility Mummies” by Santiago-Rodriguez et al. published last year in the journal Genes [1,2]. Firstly, data presented in Figure 1A are not at the species level, as claimed by Eisenhofer and Weyrich [1]. To clarify and to put results into perspective, 14 genera have been classified within the *Sphingomonadales* order alone, and a number of different operational taxonomic units (OTUs) can be classified within this order; therefore, OTUs classified as *Sphingomonadales* in the mummified guts may not necessarily represent those identified in the blank control. As stated in our study, the majority of the OTUs could not be classified at higher taxonomical levels. The limitations of being unable to classify data at the species level are intrinsic limitations within the microbiome field. One possible reason is that only one 16S region was sequenced in the study (V4 region), and stringent parameters were applied, possibly limiting our ability to resolve species-level classification. Also, it has been shown in modern and ancient microbiome studies that the taxonomic classifier can influence results [3,4,5].

Secondly, several of the bacterial orders highlighted in Figure 1A by Eisenhofer and Weyrich are bacteria that can be found in several different environments including, and not surprisingly, the human gut. *Sphingomonas* spp., for example, have been recognized as essential in maintaining human gut homeostasis [6]. Other bacteria that are present in potentially contaminating environments (i.e., soil, skin and built environments) are also members of the human gut microbiome (Table 1). Relative abundance data are shown in Table 1 as percentages. While some of these bacterial orders are present in higher relative abundances in certain individuals (i.e., *Bacillales* in mummy FI3, *Clostridiales* in mummies FI9 and FI12, and *Sphingomonadales* in mummies NASD3 and NASD14), results show that most of the bacterial orders of concern to Eisenhofer and Weyrich are present in low relative abundances, representing <1.0% of the microbiome in most of the mummies (Table 1). Notably, taxonomic data are normally presented as relative abundances, and percentages often represent numbers that are relative to the total number of reads in a sample. In addition, as noted by Eisenhofer and Weyrich, blank controls showed signals for specific bacterial OTUs. As microbiologists assessing the composition of such samples, it is our responsibility to report the OTUs present in the blank control. Various laboratories conducting ancient microbiome research have reported the presence of sequences in their blank controls as well [7,8]; thus, it is evident that many blank controls have inherent bacterial contamination [9]. In addition, detection of OTUs in the blank control can be affected by how these samples were processed. Specifically, if the blank control was processed along with the samples of interest, cross-contamination can occur. The extent of the cross-contamination can depend on how the samples were handled. On the other hand, if the blank control is processed separately from the rest of the samples, cross-contamination is less likely to occur [10]. Ideally, blank controls should be processed along with the samples of interest to provide an idea of the extent of the cross-contamination. The microbiome research community is currently discussing if OTUs present in a blank control should be removed from the sample of interest. One main reason is that a number of these OTUs present in a blank control may be part of the autochthonous microbiome of the sample of interest [11]; thus, filtering OTUs may not necessarily be a standard in microbiome research in all cases.

Thirdly, if the blank control microbiome was to significantly alter the microbiome composition, all the samples would have reflected the same level of contamination. This has been our experience when working with other low-biomass microbiomes, in which blank controls can overwhelm the resident microbiota of the sample of interest at very similar proportions across the samples, and sequences need to be discarded. The reasons for the differences in the relative abundances of the “contaminant” sequences (as stated by Eisenhofer and Weyrich) across the individuals remain unknown. However, in addition to these samples undergoing changes in water content, temperature, oxygen, and pH levels (all of which are known to affect the membership and function of microbial communities) [22,23,24,25], all of the mummies exhibited differences in terms of age, sex, diet, and comorbidities, factors that are also known to shape the microbiome [11].

**Figure 2:**
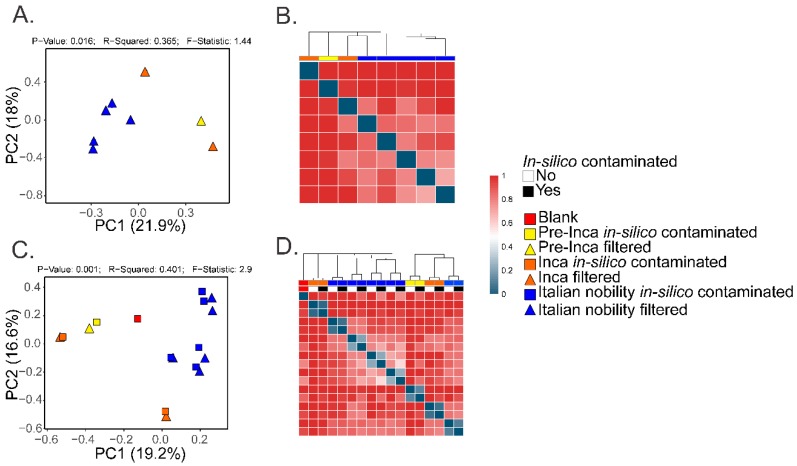
Beta diversity of the mummified gut remains. Results show the beta-diversity (Bray Curtis index) visualized as Principal Coordinate Analyses (PCoA) (**A**) and hierarchical cluster plots (**B**). Values were calculated after filtering OTUs present in the blank control from the mummified microbiome (see text). Results also show the beta-diversity (Bray-Curtis index) of the in-silico contaminated samples (see text) compared to the filtered samples. Results were visualized as PCoA plots (**C**) and hierarchical cluster plots (**D**).

**Figure 3:**
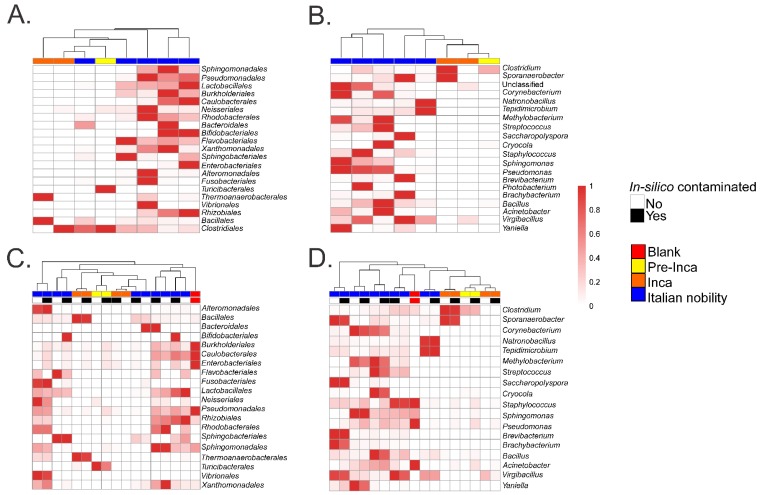
Heatmap of relative abundances of selected taxa. Results show the relative abundances of selected OTUs at the order (**A**) and genus level (**B**). Values were plotted after filtering OTUs present in the blank control from the mummified microbiome (see text). Results also show the relative abundances of selected OTUs at the order (**C**) and genus level (**D**) of the in-silico contaminated samples (see text) compared to the filtered samples.

To assess if the OTUs present in the blank control affected the interpretation of the previous results, we ran the biom summarize-table script to assess the total number of counts or sequences per sample. The pre-Inca/Inca mummies showed 78,903 (mummy FI3); 24,956 (mummy FI9); and 56,971 (mummy FI12) counts or sequences per sample. When running the script for the Italian nobility mummies, 32,086 (mummy NASD3); 44,255 (mummy NASD14); 60,266 (mummy NASD22); 24,633 (mummy NASD27); and 101,517 (mummy NASD29) counts or sequences per sample were identified. The blank control showed 3824 counts or sequences. To address if the 3824 counts or sequences identified in the blank control could affect the separation of the 16S data based on culture, we filtered the OTUs present in the blank control from the biom table of the mummies using the script filter_otus_from_otu_table.py. The total number of counts or sequences pre- and post-filtering are reported in Table 2. The number of OTUs in the blank control is also shown in Table 2. As stated, the number of OTUs in a blank control can differ depending on how it was processed. We also added the OTUs identified in the blank control to the filtered OTU table to mimic in-silico contamination. Briefly, OTU tables from the mummies and the blank control were merged using the merge_otu_tables.py script available in the Quantitative Insights Into Microbial Ecology (QIIME) package [26]. We then performed diversity and taxonomical analyses using the core_diversity_analyses.py script in QIIME. Results of the filtered samples virtually demonstrate similar dynamics as shown in our previous paper, which included non-filtered samples. Pre-Inca/Inca mummies showed lower observed Operational Taxonomic Units (OTUs) (Figure 1A) and evenness (Shannon diversity index) (Figure 1B) values compared to the Italian nobility mummies. In-silico contamination of the mummies OTU tables showed no significant differences in the alpha-diversity when looking at both observed OTUs (Figure 1C) and evenness (Shannon diversity index) (Figure 1D). Beta diversities were visualized as Principal Coordinate Analyses (PCoA) plots and showed a statistically significant separation of the data based on culture (Bray-Curtis index; the P-value, R-squared, and F-statistic values are shown (PERMANOVA test); Figure 2A). Hierarchical cluster plots of the beta-diversity (Bray-Curtis index) also showed a clear separation of the data based on culture (Figure 2B). Beta-diversity (Bray-Curtis index), visualized as PCoA plots, of the in-silico contaminated mummies OTU tables showed a separation of the data based on culture (The P-value, R-squared, and F-statistic values are shown (PERMANOVA test); Figure 2C). Hierarchical cluster plots showed that one of the Italian nobility mummies and one the Inca mummies did not cluster with the corresponding culture. This may suggest that in-silico contamination of the mummies OTU tables may affect, to some extent, the beta-diversity of certain samples. Heatmaps of the relative abundances of bacterial OTUs at the order level showed a separation of the data based on culture, except for the pre-Inca mummy (FI9), but closely resembled the Inca mummies (Figure 3A). We also performed the analysis at the genus level and noticed a separation of the data based on culture (Figure 3B). Heatmaps of the relative abundance of bacterial OTUs at the order level of the in-silico contaminated mummies OTU tables showed that two of the Italian nobility mummies microbiomes did not cluster with the rest of the Italian nobility microbiomes (Figure 3C). Similar outcomes were observed at the genus level (Figure 3D). This may suggest that in-silico contamination of the mummies OTU tables may affect, to some extent, clustering of the taxonomic data. These results support our previous study and suggest that OTUs present in a blank control may not necessarily affect the structure of a microbiome of interest.

Results from the in-silico contamination seem to support that the patterns observed in the previous article are not due to contamination of the extraction blank, and that the separation may be likely due to the differences mentioned in the original article, including dietary habits [2]. The in-silico contamination showed that a blank control may not necessarily affect the patterns observed in microbiome samples, but that any changes in observed patterns may be due by the extent of the contamination of the blank control. In addition, under any circumstances, the possibility of contamination is intrinsic to ancient DNA and microbiome work. It would be impossible to determine if the “contamination event” occurred anciently or recently; however, external artifacts are more prone to either type of contamination; in our case, specifically, the samples were obtained as they would be under surgery, and in specialized ancient DNA laboratories.

## 2. Considerations about Microbial Resilience in Ancient Samples

Numerous mechanisms, other than contamination, can explain these results, including the nature of the samples, horizontal gene transfer, sporulation, dormancy, and stress response, just to mention a few [27]. When DNA damage occurs, again, mostly upon cell death, tools such as mapDamage can aid to assess misincorporations within a particular sequence [28]. We have repeatedly stated that mapDamage provides very useful information when only one genome is being considered. In addition, cytosine deamination may be strongly influenced by sample age and temperature of collection site [29]. Utilizing mapDamage in microbiomes would also require deep sequencing, which is often costly, to correctly assess misincorporations. In fact, mapDamage analyses would have to be performed in the hundreds of different species genomes present in a sample. Failing to find misincorporations in sequences present in microbiome samples that have failed to reach enough coverage is not necessarily evidence of contamination.

In terms of self-plagiarism mentioned by Eisenhofer and Weyrich, the mapDamage original results were published in FEMS Microbiology Letters [30]. The journal is currently part of the Oxford Academic journals, and as part of the copyright agreement with Oxford University Press, we have retained the right to use all or part of the article and abstract, including the supplementary figures after publication.

As we have previously done in numerous occasions, we thank our concerned colleagues for their comments. However, science may be better served by possible collaborations and publishing original research rather than after-the-fact criticism. As we obtain data on ancient DNA, we continue to better understand the microbiome of ancient samples, taking into consideration what we know so far about modern microbiomes, and the numerous mechanisms of microbial resilience and DNA repair. We will continue to report our findings on data obtained following strict guidelines to avoid contamination and will keep carrying out original research that will result in a better understanding of ancient ethnic groups despite possible shortcomings.

## Figures and Tables

**Table 1:** Abundances (%) of selected bacterial taxa at the order level in the pre-Inca/Inca (FI3, FI9, and FI12) and Italian nobility mummies (NASD3, NASD14, NASD22, NASD27, and NASD29), and the blank control. The percentages are relative to the total number of reads in each sample.

Order (16S Data)	Mummy									Presence in Human Gut References
	FI3	FI9	FI12	NASD3	NASD14	NASD22	NASD27	NASD29	Blank	
*Sphingomonadales*	<0.01	0.20	0.02	35.14	14.934	0.51	8.74	0.22	3.01	[6,12]
*Pseudomonadales*	<0.01	0.15	<0.01	6.35	6.66	0.54	7.27	0.17	20.42	[6,13,14]
*Lactobacillales*	<0.01	0.12	0.01	2.87	2.76	3.65	5.98	0.34	0.10	[12,13,15]
*Burkholderiales*	<0.01	<0.01	0.00	2.52	0.67	0.90	1.32	0.08	15.43	[12,16]
*Caulobacteriales*	<0.01	0.01	0.00	1.08	0.24	0.04	1.69	0.04	6.90	[17]
*Neisseriales*	<0.01	0.02	<0.01	<0.01	0.08	<0.01	<0.01	<0.01	0.00	[13,18]
*Rhodobacterales*	<0.01	0.00	0.00	0.02	0.05	0.01	0.01	<0.01	0.05	[12,19]
*Xanthomonadales*	<0.01	0.00	0.00	0.15	0.11	0.02	0.02	0.01	0.05	[13,20]
*Sphingobacteriales*	<0.01	0.00	0.00	0.00	<0.01	0.19	0.05	0.02	0.03	[12,21]
*Enterobacteriales*	<0.01	0.00	<0.01	0.10	0.01	<0.01	0.25	0.00	7.01	[12,13,21]
*Rhizobiales*	0.04	0.47	0.12	18.9	8.93	0.39	44.94	0.65	5.36	[12,13]
*Bacillales*	96.82	0.36	0.01	7.30	29.66	14.81	2.91	23.83	9.91	[13,15,21]
*Clostridiales*	0.04	97.65	99.6	4.10	19.79	34.00	9.49	73.93	17.34	[12,13]

**Table 2:** Sequence counts per sample prior and after filtering of operational taxonomic units (OTUs) present in blank control. Sequence counts were obtained using the biom summarize-table script.

Sample	Counts Prior Filtering	Counts after Filtering
Blank	3824	NA
FI3	78,903	78,881
FI9	24,956	24,495
FI12	56,971	56,846
NASD3	32,086	14,768
NASD14	44,255	24,740
NASD22	60,266	55,727
NASD27	24,633	13,392
NASD29	101,517	100,028
